# Interventions in Chinese Undergraduate Students’ Mental Health: Systematic Review

**DOI:** 10.2196/38249

**Published:** 2022-06-15

**Authors:** Yi Shan, Meng Ji, Wenxiu Xie, Rongying Li, Xiaobo Qian, Xiaomin Zhang, Tianyong Hao

**Affiliations:** 1 School of Foreign Studies Nantong University Nantong China; 2 School of Languages and Cultures University of Sydney Sydney Australia; 3 Department of Computer Science City University of Hong Kong Hong Kong China; 4 School of Artificial Intelligence South China Normal University Guangzhou China; 5 School of Computer Science South China Normal University Guangzhou China; 6 Department of Linguistics Macquarie University Sydney Australia

**Keywords:** systematic review, intervention, mental health, depression, anxiety, stress, Chinese undergraduate students

## Abstract

**Background:**

Over 30% of university students from 8 countries were afflicted with mental distress according to a World Health Organization survey. Undergraduate students in increasing numbers in China have also been reported to suffer from different mental problems. Various psychological distresses significantly impact their academic and daily life, thereby causing role impairments and unsatisfactory academic achievements. While the prevalence of, diverse underlying factors for, and interventions of social support in college students’ mental health have extensively been investigated in China, there is no study exclusively focusing on the impact of interventions on their psychological well-being.

**Objective:**

The aim of this review was to identify and synthesize the interventions in the mental health concerns of Chinese undergraduate students studying in China reported in the literature to inform educational authorities, college and university management, students’ affairs counselors, and mental health providers.

**Methods:**

We performed a systematic review and reported the research findings of previous studies according to the protocol of the PRISMA (Preferred Reporting Items for Systematic Reviews and Meta-Analyses) 2020 statement. First, based on the predefined search strategy, keyword searches were performed in the PubMed and ProQuest databases to retrieve relevant studies. Subsequently, we screened the candidate articles based on predefined inclusion and exclusion criteria. Finally, we analyzed the included papers for qualitative synthesis.

**Results:**

We retrieved a total of 675 studies from the PubMed and ProQuest databases using the search strategy on March 15, 2022. Among these candidate studies, 15 that were not written in English, 76 duplicates, and 149 studies of other document types were removed before screening. An additional 313 studies were excluded in the screening process, with 73 articles ruled out for being not relevant to interventions, not related to mental health, or not focused on undergraduate students in the full-text review. As a result, 49 papers were eligible and included in this systematic review. In the qualitative synthesis, we divided the interventions reported in the selected studies into two categories: (1) social support from government authorities, university authorities, students’ affairs counselors and teachers, family members, health care authorities and professionals, and the media (various online platforms), and (2) various coping strategies adopted by undergraduate students themselves. We identified further research on mental health interventions that may be delivered by digital medical platforms, conversational agents (eg, chatbots), and researchers.

**Conclusions:**

This was the first systematic review of interventions to address the mental health concerns of Chinese undergraduate students studying in China. The categorization of reported interventions and the identification of new intervention channels can effectively inform stakeholders. Interventions for undergraduate students’ mental health is a research topic worth further investigation.

## Introduction

### Background

Over 30% of university students from 8 countries were afflicted with mental distress according to a World Health Organization survey [1]. Undergraduate students in increasing numbers in China have also been reported to suffer from different mental problems [2], including depression, compulsion, anxiety, and interpersonal sensitivity [3-6]. Students experienced different degrees of depressive symptoms in different parts of China, with an incidence of 9.7% in Eastern and Western China, 11.7% in Harbin, 11.8% in 6 universities in Wuhan, 16.8% in Anhui, and 32.82% in Western Liaoning [7-11]. Transitioning from adolescence to adulthood while leaving home to attend colleges requires facing many challenges independently [12], which causes an increase in symptoms of depression, anxiety, and stress [13-15]. Various psychological distresses significantly impact the academic and daily lives of students, thereby causing role impairments and unsatisfactory academic achievements [16-18].

Given the prevalence of mental disorders in 28.4% of Chinese college students [19], studies have been performed to identify the various underlying contributing factors such as interpersonal relationships [6]; multiple factors from individuals, families, schools, and society [12]; gender and income [20,21]; academic stress and load, financial difficulty, departure from home, unstable family, and bullying on campus [3,4,22,23]; personal behaviors and social settings [24]; and lack of physical activities [25]. Pinpointing these factors contributing to college students’ mental distress facilitates developing effective mental health interventions to reduce potential adverse impacts on their psychological well-being. Mental health promotion and prevention are needed to improve the mental health condition of college students who are especially vulnerable to pressure and other mental health issues [26], which will contribute to their overall well-being [27]. While the prevalence of, diverse underlying factors for, and interventions of social support in college students’ mental health have been extensively investigated in China, there is no study exclusively addressing the interventions for their mental health concerns.

### Interventions in the Literature

Social support has proven to be one of the most critical and effective interventions to mitigate mental health risks imposed on college students [28-30]. Social support is a form of mutual communication and connection network, including emotional support, instrumental support, and informational support [31]. Such support has been strongly associated with mental health among college students [32]. When obtaining robust social support from friends, family, and teachers, university students had better mental health to sustain themselves against crises and stress [32]. Social support was proven to moderate the relationship between stress and depression [33]. High levels of social support could buffer mental health concerns [34].

Various therapies are also effective mental health interventions. Several therapies have showed effects on par with those of pharmacological treatment [35], including psychotherapy [36], interpersonal therapy [37], problem-solving therapy [38], supportive therapy [39], psychoeducation [40], and exercise/physical activity [41].

Universities have traditionally been providing mental health services in clinical settings, such as face-to-face individual or group-based consultations [42]. However, the available resources of many universities are too limited to support comprehensive approaches to students’ mental health, and students are frequently unwilling to visit traditionally structured student counseling centers for help [42,43]. Therefore, it is necessary to identify effective mental health interventions that can be delivered to students in virtual settings and cover the spectrum of interventions from prevention to treatment [44].

Mowbray et al [44] developed internet-based interventions that were designed to promote mental health help-seeking, including a mental health literacy/destigmatization intervention, a feedback intervention, and a help-seeking list intervention. Web-based depression and anxiety interventions have been confirmed to be effective for treating common mental disorders [45,46].

### Objective

The aim of this review was to identify and synthesize the interventions addressing the mental health concerns of Chinese undergraduate students studying in China reported in the literature. The synthesized interventions are expected to inform stakeholders, including educational authorities, college and university management, students’ affairs counselors, and mental health providers.

## Methods

### Study Design

To analyze and synthesize the interventions focused on the mental health of Chinese undergraduate students studying in China, we performed a systematic review and reported the research findings according to the protocol of the PRISMA (Preferred Reporting Items for Systematic Reviews and Meta-Analyses) 2020 statement [47]. We applied keyword searches to retrieve publications related to this research topic in two databases (PubMed and ProQuest), and screened the candidate articles based on the predefined inclusion and exclusion criteria. Finally, we analyzed the included papers for qualitative synthesis.

### Search Strategy

We searched the PubMed and ProQuest databases to identify relevant studies. Based on the studies mentioned in the Introduction, we defined undergraduates’ mental health concerns as issues related to depression, anxiety, stress, and disorder. Drawing on these keywords, we designed the following search strategy for this review: ((college student [Title/Abstract]) OR (university student [Title/Abstract]) OR (student [Title/Abstract])) AND ((digit* [Title/Abstract]) OR (online [Title/Abstract])) AND ((mental health [Title/Abstract]) OR (mental disorder [Title/Abstract]) OR (depression* [Title/Abstract]) OR (anxiety [Title/Abstract]) OR (disorder [Title/Abstract]) OR (stress [Title/Abstract])) AND (intervention) AND ((Chinese) OR (non-English) OR (Chinese-speaking)). Considering that many relevant articles were not published in peer-reviewed journals, we considered both peer-reviewed and nonpeer-reviewed articles in this systematic review. We did not impose any restrictions on the publication date of the candidate articles to retrieve all papers related to this topic. On March 15, 2022, we retrieved the candidate studies from the PubMed and ProQuest databases using the above search strategy. The keywords of the strategy were searched in the titles and abstracts of the candidate articles.

### Study Selection Criteria

Papers that were not written in English were excluded because translation of the articles was not feasible or reliable. Additionally, we only included journal articles and excluded other article types (eg, review papers, letters, reports, and editorials).

This systematic review focused on the interventions in the mental health of Chinese undergraduate students in China. Therefore, the following criteria had to be satisfied in the selection of eligible papers: (1) the target population is undergraduate students; (2) the undergraduate students are Chinese who study in colleges and universities in China; and (3) the candidate articles need to be related to interventions for undergraduate students’ mental health or mental disorder problems, including depression, anxiety, and stress. Articles that were focused on undergraduate students’ mental health or mental disorder problems but involved no interventions, and those focused on interventions addressing the mental health or mental disorder problems of Chinese undergraduate students studying in countries other than China were excluded from this review.

### Screening and Article Selection

We used Microsoft Excel to collect and manage the data of the candidate papers, including author, year of publication, country, target population (participants), study design/method, interventions, and limitations. The screening of the eligible studies was performed in the following two steps. First, two reviewers (YS and YC) reviewed the titles and abstracts of the candidate articles, excluding those that were not related to interventions addressing the mental health concerns of Chinese undergraduate students studying in China. Papers whose eligibility was unclear were retained for the full-text review. Second, six reviewers (YS, YC, XQ, RL, XW, and TL) reviewed the full texts of the remaining articles independently. Any controversies were resolved through discussing and consulting with two additional authors (MJ and WX) to make final decisions at the panel meeting of all members of the research team.

### Data Extraction

We extracted data in light of our research objective to collect key information from eligible articles. The data extraction was performed by six researchers (YS, YC, XQ, RL, XW, and TL) independently, and reviewed and cross-checked by two researchers (MJ and WX). Any discrepancies were addressed through a consensus discussion at the panel meeting of all members of the research team.

### Data Analysis and Synthesis

It was not feasible to conduct a meta-analysis owing to the expected variety of study designs, mental health interventions, and study limitations. As such, a descriptive analysis was carried out to summarize the data extracted from the included papers.

## Results

### Search Results

**Figure 1:**
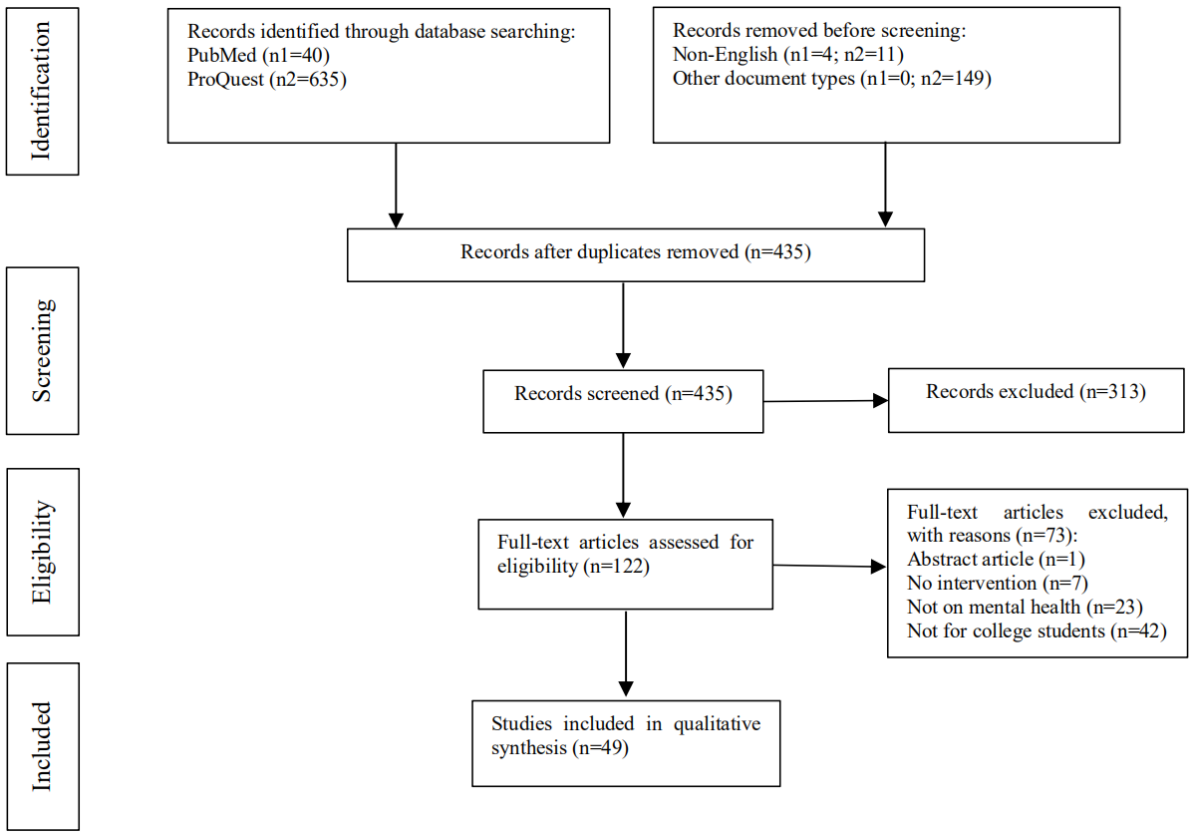
PRISMA (Preferred Reporting Items for Systematic Reviews and Meta-Analyses) flowchart of the selection of eligible studies.

We retrieved a total of 675 studies from the PubMed and ProQuest databases using the search strategy. Among these candidate studies, 15 that were not written in English, 76 duplicates, and 149 studies of other document types were removed before screening. An additional 313 studies were excluded in the screening process, with 73 articles ruled out for being not relevant to interventions, not related to mental health, or not including undergraduate students in the full-text review. As a result, 49 papers were deemed to be eligible and included in this systematic review. The PRISMA flowchart of the screening and reviewing processes is provided in Figure 1.

### Characteristics of Included Studies

The following items of the extracted data were obtained: publication information (name of authors, country, and publication year), target population (participants), study design (research methods), interventions, and limitations. Table S1 in Multimedia Appendix 1 describes these characteristics of the 49 included studies.

Interventions in the mental health of Chinese undergraduate students studying in China have only recently attracted scholarly attention, as evidenced by the fact that all 49 included studies [2,12,26,48-93] were published between 2020 and 2022, including 11 (22%) published in 2020, 33 (67%) published in 2021, and 2 (4%) published in 2022. This indicates the worsening mental health condition of this particular population in recent years, especially in the context of the repeated resurgences of the COVID-19 pandemic, which increased the stress of college students and exposed them to new frustrating stressors that caused various mental health concerns [2]. Given college students’ high stress and anxiety levels during the COVID-19 pandemic [88], 35 (71%) of the 49 studies [2,12,48,49,52-58,61-64,67-69,72-77,81-89,91,92] investigated the COVID-19–induced mental health concerns of Chinese undergraduate students and proposed specific interventions in response to the severity of the psychological impact of COVID-19 [87].

The included studies were primarily conducted as cross-sectional surveys via online questionnaires. The main limitation of these studies is that self-reported online questionnaires are likely to result in a certain degree of recall bias and response bias due to the stigma attached to mental health conditions.

### Undergraduates’ Mental Health Concerns

In the 49 included studies, we identified the following forms of mental health concerns: depression, anxiety, stress, interpersonal sensitivity, fear, distress, psychological disorders, self-harm/suicidal, insomnia, obsessive interpersonal sensitivity, trauma, negative emotions, and insecurity.

### Categorization of Mental Health Interventions

#### Overview

The interventions proposed in the included studies can be divided into two broad categories: social support and coping strategies. Social support was provided by government authorities [49,52-54,61,62,68,72,84,86-88]; university authorities [12,48,50,51,53-55,57-59,62,64,65,67,69,91,93]; students’ affairs counselors and teachers [54,75,83-85,87,93]; family members [52,54,57,83,94]; health care authorities and professionals [52,63,66,75,77-79,87]; researchers [70,85]; and media-, internet-, and smartphone-based interventions [26,53,55,56,60,71,73,75,77,80,81]. Positive coping strategies were adopted by undergraduate students themselves [52,82,83,85,86,89,92,93]. In addition, negative coping strategies were also reported in a few studies [52,59,74,75,89]. All of the interventions are listed in Table 1.

**Table 1:** Interventions reported in the 49 included studies.

Reference	Interventions, conclusions, and recommendations
Huang et al [2]	Active coping strategies helped improve their psychological well-being; family support was particularly important for maintaining mental health and ameliorating mental health challenges in this major health crisis; suitable psychointervention, routine screening for risk behaviors, and provision of further social support are needed for undergraduate students in the COVID-19 pandemic or other emergency public health events
Lei et al [12]	Universities should develop a care culture and environment that supports the life adjustment of college students, promotes cultural and sports activities, and facilitates the expansion of social networks; mental health education and psychological counseling services should be strengthened, including hotlines offering timely help to address students’ urgent needs; early detection and effective management of mental health problems can effectively reduce serious mental health disorders
Mak et al [26]	Internet-based cognitive behavioral and mindfulness training programs are effective, which can be easily incorporated into existing service provision portfolios that promote mental health and reduce psychological distress to ultimately promote mental health among college students and young working adults
Yu et al [48]	Screening for ACEs^a^, and strength-based, trauma-informed interventions on fostering resilience are needed to promote mental well-being among Chinese young adults
Zhang et al [49]	In practical interventions, authorities (eg, governments and universities) should first focus on improving efficacy appraisal by providing psychological support to gain the trust of college students so that they believe in and comply with scientific prevention and control measures. By inviting psychiatrists to deliver lectures, authorities can reasonably and effectively enhance the public information of COVID-19–related knowledge and scientific prevention and control measures.
Shen et al [50]	It is important to address ADHD^b^ symptoms among students with anxiety; it is of importance to screen medical students for anxiety disorders to better promote the mental health and well-being of this population and better prevent suicidal behaviors
Sze et al [51]	Given its associations with negative emotions and other aspects of health, screening and management of EE^c^ may improve multiple areas of health and well-being
Zhao and Zhou [52]	It is critical for policymakers, public health agencies, parents, psychologists, and health care staff to remain sensitive to the potential negative consequences of ubiquitous social media exposure; the general public, especially those who have been directly or indirectly traumatized by COVID-19, could be advised to avoid excessive social media use and learn effective emotion regulation strategies (eg, reappraisal) to reduce negative emotions induced by news coverage
Yu et al [53]	The government can open free psychological hotline consultations to help college students solve their psychological problems; the media should release correct information in a timely manner and prevent the spread of rumors; universities can actively organize health education activities and encourage college students to arrange their time reasonably and take the initiative to find a suitable way to relieve stress during home quarantine
Li and Peng [54]	Adopting positive coping strategies may enhance social support that in turn relieves anxiety. The effect of social support, especially family and counselor support, can decrease anxiety in coping with the COVID-19 pandemic cognitively and behaviorally. Policymakers and school administrators should encourage meaningful communication between family members and activate effective counseling services to maintain positive mental health
Wang et al [55]	Reducing SNS^d^ addiction and mental problems by conducting interventions using cognitive behavioral approaches; screening for and addressing excessive SNS use are needed to prevent SNS addiction and mental distress among young people.
Sit et al [56]	Evidence-based digital mental health interventions
Liang et al [57]	University campuses should develop and implement effective screening procedures to closely monitor students’ exposure to stressors and mental health status; psychological intervention programs should be designed to address fear and fully utilize psychological assistance hotlines to help college students better adjust themselves; performing psychological help-seeking intervention, strengthening the dissemination of mental health knowledge, and improving the level of mental health perception are effective ways to improve help-seeking attitudes and increase the probability that college students will seek psychological help
Nurunnabiet al [58]	University authorities should be aware of students’ coping strategies. In particular, students who live without parents or relatives should be taken care of properly during the outbreak. To help students cope with the mental pressure, university authorities may consider arranging or organizing programs such as an online experience-sharing competition, and encourage students by offering rewards or financial aids. Required food and health care materials should be supplied to ensure the students’ safety
Wu et al [59]	Sleep hygiene, mobile phone and internet use hygiene, mental health education courses, professional psychological counseling, and other interventions should be considered and implemented. Appropriate interventions that target problematic smartphone use could potentially reduce anxiety and depression levels, which will in turn provide a buffer against the negative impact of poor sleep quality on eating disorder symptoms
Chen et al [60]	Web-based intervention for subclinical depression (MoodBox) informed by evidence-based psychological interventions, including CBT^e^, IPT^f^, and mindfulness meditation
Yu et al [61]	Various cognitive, behavioral, and psychosocial responses to COVID-19 showed both direct and indirect effects (via mental distress due to COVID-19) on depression. Thus, interventions to improve such multidimensional factors might reduce mental distress during the initial COVID-19 outbreak period
Zhang et al [62]	Relevant education and psychological counseling to parents during the outbreak to help them understand their children’s mental state, with universities providing psychological counseling and psychological interventions to students, focusing on college students who are most severely affected by the epidemic
Li et al [63]	Recommends providing long-term psychological services for students; the results could help health care professionals identify college students at high risk of mental health problems so that appropriate interventions can be targeted against them
Tang et al [64]	Recommends providing psychological interventions for quarantined college students to help them reduce fear and improve sleep duration. Universities need to consider planning acute and long-term psychological services for more vulnerable students, graduates, and students living in the most severely affected areas
Zhou et al [65]	One week of positive mental imagery training can help to improve negative emotions and anxiety in depression; further exploration of this training program is suggested
Shen et al [66]	Providing mental health care and counseling services to students of high-risk groups in medical schools; the early diagnosis and treatment of ADHD may have a suicide prevention effect
Li et al [67]	Parents strengthen communication with their children and provide psychological support to their children. Universities carry out relevant online mental health courses and implement psychological intervention measures to improve students’ psychological adaptability
Yang et al [68]	The government, school administrators, and society strengthen operability research to provide coping strategies, implement psychological interventions, and conduct relevant training
Liu et al [69]	Universities should adopt a web-based PPI^g^ to improve the mental health of college students
Carciofo [70]	Longitudinal studies of these variables may establish causal relationships and may inform interventions to treat psychological distress and disorders
Yen et al [71]	Depressed college students have less hostility after entering the internet, suggesting that the internet as a useful medium to provide treatment for people with depression
Zheng et al [72]	Recommends adequate social support and long-term targeted psychological interventions for college students. More serious mental health problems seen among fourth-year students, proposing to specifically increase their employment opportunities and develop mental health rehabilitation programs
Song et al [73]	Online or smartphone-based psychoeducation and psychological interventions that will also reduce the risk of virus transmission by foregoing face-to-face therapy
Tao et al [74]	Unsupervised, self-initiated interventions against mental and sleep disorders of students can lead to more disastrous outcomes
Jia et al [75]	Public health education from health authorities in various governments is needed for dissemination of the importance of preventive measures during COVID-19. Psychological health services should be implemented to alleviate the adverse effects of this pandemic under national social distancing. Psychological interventions could also be carried out through online platforms under national social distancing during COVID-19. Teachers should also pay attention to strengthening the dissemination of COVID-19 knowledge and preventive measures to reduce the level of anxiety and depression in the student population
Dun et al [76]	Interventions to decrease sedentary time and improve mental health may be warranted to mitigate weight gain during the lockdown period and reverse the weight gain in youth after the COVID-19 pandemic
Yu et al [77]	Grief counseling and online sacramental ceremonies should be implemented for this group to prevent negative emotional difficulties; mindfulness meditation and CBT can reduce students’ anxiety and depression
Pan and Zhuang [78]	Integration of cognitive behavioral intervention and adventure training in a class setting might be an effective and feasible approach for the mental health counseling of university students
Auyeung and Mo [79]	PPI
Zhao et al [80]	Mediating effect of online social support was stronger among college students with lower perceived social support than those with higher perceived social support
Xin et al [81]	Online brief interventions need to be made available, including screening of mental distress, counseling hotlines, emotional regulation and coping skills, and promotion of positive psychology
Liang et al [82]	Compared to meeting no guidelines, meeting the sleep guideline (alone or in combination with other guidelines) was associated with significantly lower levels of depression and anxiety; meeting both SB^h^ and MVPA^i^ guidelines was also associated with a significantly lower level of depression. Hence, meeting more guidelines, especially adhering to a healthy sleep routine, may play an important role in promoting the mental health of young adults
Sun et al [83]	Perceived available peer support negatively contributed to depressive symptoms. Both negative and positive indicators of emotional well-being mediated the association between perceived available peer support and depressive symptoms, and advanced the practical needs for preventive efforts and accessible care to support the psychological and emotional needs of young people during the COVID-19 pandemic.
Li et al [84]	Over 50% of the participants had obvious fear and anxiety symptoms at 61.64% and 58.39%, respectively. Conformity (49.49%), invulnerability (26.11%), insensitivity (21.49%), and rebelliousness (12.41%) symptoms also appeared. Senior students experienced more anxiety than freshmen. Psychological symptoms (except for insensitivity) had no significant difference with respect to gender, residence, and annual household income in one-way analysis of variance
Zhu et al [85]	Association between mental health and emotion regulation, which will help direct a psychological intervention that relieves these issues during the pandemic
Zhuo et al [86]	Back-to-school students who are certain and uncertain that COVID-19 will rebound again were significantly more anxious and depressed than those with optimistic attitudes. Government departments should pay high attention to the mental health problems evoked by intolerance of uncertainty (IU). Social support as a moderator could buffer the relationship between IU and mental health, including anxiety and depression during unprecedentedly uncertain times
Li et al [87]	Mental health services reducing PTSD^j^ should be provided; students who have lost loved ones and suffered family financial loss should be given particular care
Zhan et al [88]	Education departments should attach great importance to the mental health of college students, and it is necessary to provide precise psychological interventions for groups experiencing greater pressure levels and marked anxiety and depression
Ding et al [89]	Three coping styles were all significantly correlated with psychological distress in Chinese college students during the early stage of the COVID-19 pandemic. Adaptive emotion-focused coping was negatively associated with perceived stress and psychological distress. Emotion-focused coping was positively associated with perceived stress and distress. Individuals who use specific reactive emotion-focused coping strategies more often, such as focusing on emotions, denial, seeking emotional social support, and disengaging, experience more stress.
Zhou et al [90]	Intercultural cooperation should be promoted to develop a cross-culturally valid concept of stigma against psychological help that could be used as the basis for intercultural comparison and developing interventions to reduce stigma
Liang et al [91]	Guiding postgraduate students to correctly understand their mental health status and individual differences in mental tolerance, and encouraging postgraduate students to seek help if they experience psychological problems so as to help them adjust their goals and plans according to reality, and avoid the development of other problems such as PTSD; establishing an early warning system for the mental health of postgraduate students during the pandemic and improve online and offline psychological counseling service systems; considering the characteristics and situation of different postgraduate groups for postgraduate student management to develop targeted mental health education programs and adopting objective measures, so as to improve postgraduate mental health and nurture both physical and mental health to facilitate China’s modernization; developing and maintaining conditions to improve the communication between postgraduate students and advisors during the pandemic and create a new postgraduate guidance mode to relieve the psychological problems of postgraduate students
Wen et al [92]	Enhancing positive self-beliefs such as hope and self-efficacy helps to buffer the effects of insecurity on stress. Physical and psychological exercises that enhance hope can be effective interventions to help university students buffer the impacts of insecurity and alleviate stress during the outbreak. Improving positive self-beliefs can help to relieve the pressure of university students during the outbreak. University students can improve their self-efficacy by participating in movement-based courses, including Pilates and Tai Chi, so as to improve their positive mood and relieve stress. They can also effectively improve their hope levels by setting personal goals and conducting goal-pursuit exercises, which can also contribute to the reduction of insecurity and stress
Lin et al [93]	Dental schools and educators promote stress-coping strategies and modify teaching curricula to reduce students’ stress. Stress management efforts such as time management, encouragement from advisors, and regular exercise are recommended

^a^ACE: adverse childhood experience.

^b^ADHD: attention deficit and hyperactivity disorder.

^c^EE: emotional eating.

^d^SNS: social networking site.

^e^CBT: cognitive behavioral therapy.

^f^IPT: interpersonal psychotherapy.

^g^PPI: positive psychology intervention.

^h^SB: sedentary behavior.

^i^MVPA: moderate-to-vigorous physical activity.

^j^PTSD: posttraumatic stress disorder.

#### Social Support

##### Main Categories

In the 49 included studies, we identified various types of social support from government authorities; university authorities; students’ affairs counselors and teachers; family members; health care authorities and professionals; researchers; and the media-, internet-, and smartphone-based interventions.

##### Government Authorities

Governments need to join hands with school administrators and various social parties to strengthen feasibility research to offer coping strategies, perform psychological interventions, and conduct relevant training [68]. Specifically, government authorities at all levels need to (1) specially improve efficacy appraisal through providing psychological backup for undergraduate students, by inviting psychiatrists to deliver relevant lectures [49]; (2) offer free psychological counseling via hotlines to help undergraduate students solve their psychological problems [53]; (3) develop interventions to improve undergraduate students’ various cognitive, behavioral, and psychosocial responses to public health emergencies such as COVID-19 [61]; (4) provide parents with relevant education and psychological counseling to help them understand their children’s mental state [62]; (5) advocate adequate social support and long-term targeted psychological intervention to provide more employment opportunities and develop mental health rehabilitation programs for the fourth-year undergraduate students who suffered more mental problems [72,84]; (6) pay special attention to mental health concerns induced by intolerance of uncertainty [86]; and (7) particularly care for students who have lost loved ones and experienced family financial losses [87].

Policymakers and public health agencies need to (1) be sensitive to the potential adverse effects of omnipresent exposure to social media [52] and (2) encourage effective communication among family members and activate effective psychological counseling services [54].

Education departments ought to provide precise psychological interventions for those suffering greater pressure and marked anxiety and depression among undergraduate students [88].

##### University Authorities

Previous studies proposed university authorities to make the following interventions in undergraduate students’ mental health concerns, including (1) developing a caring culture and ambience, which backs up undergraduate students to make life adjustments, promoting cultural and sports activities, facilitating the expansion of social networks, strengthening mental health education and counseling (eg, hotlines providing timely help for those in urgent need), early detection and effectively managing mental health concerns [12]; (2) screening for adverse childhood experiences and providing strength-based, trauma-informed interventions on fostering resilience [48]; (3) screening for anxiety disorders [50,57]; (4) screening and managing of emotional eating [51]; (5) organizing health education activities, and encouraging undergraduate students to arrange their time reasonably and find a proper approach to alleviate stress [53]; (6) encouraging timely, effective communication between family members [54]; (7) conducting interventions using cognitive behavioral approaches and screening for and addressing excessive social networking service use [55]; (8) developing psychological help-seeking interventions, strengthening the dissemination of mental health knowledge, and improving the level of mental health perception to improve help-seeking attitudes and increase the probability of seeking psychological help [57]; (9) being aware of undergraduate students’ coping strategies, organizing programs such as an online experience-sharing competition, encouraging students by offering rewards or financial aids, and supplying necessary food and health care materials [58]; (10) implementing various interventions such as sleep hygiene, mobile phone and internet use hygiene, mental health education courses, and professional psychological counseling [59,62,64,67,91]; (11) providing positive mental imagery training [65]; (12) offering relevant online mental health courses [67]; (13) adopting a web-based positive psychology intervention [69]; (14) establishing an early warning system for mental health concerns, guiding students to correctly understand their mental health status and individual differences in mental tolerance while encouraging them to seek help if necessary, developing targeted mental health education programs, and creating a new guidance mode to improve the communication between students and counselors [91]; and (15) promoting stress-coping strategies [93].

##### Students’ Affairs Counselors and Teachers

Student counselors need to assume the responsibilities to provide support and positive coping strategies [54,93] along with psychological health services [75,85], in particular accessible care [83] for senior students who experienced more anxiety [84] and students who have lost loved ones and suffered family financial loss [87], emotion regulation guidance [85], and encouragement [93]. Teachers should pay close attention to disseminating COVID-19 knowledge and preventive measures [75], and modify teaching curricula [93] to reduce undergraduate students’ academic pressure in the face of public health emergencies and natural disasters.

##### Family Members

One study argued that the role of family support in maintaining undergraduate students’ mental health must be emphasized [94]. We found 4 studies reporting interventions delivered by family members. Family support can effectively decrease anxiety cognitively and behaviorally [54]. Therefore, parents need to strengthen communication with their children and provide psychological support for them [57], provide accessible care to support the psychological and emotional needs of their children [83], and maintain sensitivity to the potential negative consequences on their children brought about by ubiquitous social media coverage [52].

##### Health Care Authorities and Professionals

Health care professionals should identify, diagnose, and treat undergraduate students at high risk of mental health concerns early so that proper interventions, including long-term psychological services, can be tailored for them [63,66]. Grief counseling, mindfulness meditation, and cognitive behavioral therapy are recommended to reduce students’ anxiety and depression [77], along with positive psychological intervention [79], and authorities should remain sensitive to the potential negative consequences of ubiquitous social media exposure [52].

Health authorities should provide public health education, implement psychological health services online and offline [75], integrate cognitive behavioral intervention and adventure training in a class setting [78], and offer mental health services reducing posttraumatic stress disorder [87].

##### Researchers

Researchers need to conduct longitudinal studies of morning affect, eveningness, and amplitude distinctness to establish causal relationships between these factors and negative emotionality, and thus inform interventions to treat psychological distress and disorders [70]. They should also study the association between mental health and emotion regulation to help direct psychological interventions [85].

##### Media-, Internet-, and Smartphone-Based Interventions

Evidence-based digital mental health interventions were found to be useful in improving mental health concerns [56,60], because depressed undergraduate students became less hostile when logging onto the internet, suggesting the internet as a useful medium to provide treatment for people with depression [71]. In the context of face-to-face intervention delivery hindered by the COVID-19 pandemic, internet-delivered interventions such as internet-delivered cognitive behavioral therapy can be considered to address effects of social networking service addiction on the mental health status of Chinese university students [55]. It is effective to use internet-based cognitive behavioral and mindfulness training programs, which can easily be integrated into existing service provision portfolios that promote mental health and reduce psychological distress to promote the mental health of undergraduate students [26]. Online brief interventions need to be made available, including screening of mental distress, counseling hotlines, emotional regulation and coping skills, and promotion of positive psychology [60,73,75,81], whose mediating effects were proven to be stronger among undergraduate students with lower perceived social support [80]. Online sacramental ceremonies should be implemented to prevent negative emotional difficulties [77]. The media need to release correct information in a timely manner and curb the spread of rumors [53], which may aggravate the mental health concerns of psychologically vulnerable undergraduate students.

#### Coping Strategies

A recent study mentioned positive coping strategies as intervention measures to help improve college students’ psychological well-being [2]. Some of the 49 included studies reported positive coping strategies, including regulating emotions effectively [52,85]; meeting the sleep, sedentary behavior, and moderate-to-vigorous physical activity guidelines [82]; developing optimistic attitudes [86]; adopting problem-focused and adaptive emotion-focused coping [89]; enhancing positive self-beliefs (eg, hope and self-efficacy) and physical and psychological exercises [92]; and managing stress through time management and regular exercise [93]. Moreover, undergraduate students need to provide mutual peer support and accessible care, because perceived peer support and care alleviated depressive symptoms and met young people’s psychological and emotional needs [83]. All of these positive coping strategies were proven to be effective in promoting undergraduate students’ mental health [82].

However, negative coping should be avoided, which can lead to more disastrous consequences [75]. The reported negative coping strategies include excessive use of social media, which can be counteracted with emotion regulation (eg, reappraisal) to reduce negative emotions induced by news coverage [52]; problematic smartphone use [59]; unsupervised, self-initiated intervention [74]; and maladaptive emotion-focused coping [89].

## Discussion

### Principal Findings

The great uncertainty about the pandemic, the abrupt transition to and participation in online classes, and the COVID-19–related impacts on life all frequently contributed to the increased stress and anxiety of undergraduate students [95-97], in addition to their inability to tackle problems concerning interpersonal relationships, academic challenges, and career development due to the lack of life and social experience [98,99]. Therefore, 35 (71%) of the 49 included studies that investigated Chinese undergraduates’ mental health concerns induced more or less by the COVID-19 pandemic. The proposed interventions to counteract the severe psychological influence of the pandemic were synthesized into two broad categories: social support and coping strategies. Social support has proven to be an effective protective factor for mental health in previous studies [100-103]. Social support means providing practical help, emotional backup, and information assistance by those around individuals in mental distress [104]. Social support was found to be negatively correlated with adverse mental health outcomes (anxiety, depression, and insomnia), which were aggravated by COVID-19–induced intolerance of uncertainty [86]. Social support also served as a moderator buffering the relationship between intolerance of uncertainty and mental health, including anxiety and depression [86].

Coping strategies have proven to be effective protective factors for mental health in previous studies [100-103]. All of these interventions are crucially important in the context of the COVID-19 pandemic and other future public health crises or natural disasters, which can cause long-term mental disorders in various populations [99]. Therefore, the effective mental health interventions in the forms of various social support and coping strategies reported in the 49 included studies can surely shed light on the interventions in undergraduate students’ mental health issues.

Moreover, we identified some mental health interventions that were not reported in the included studies but are potentially effective and robust, including those delivered by digital medical platforms, conversational agents (eg, chatbots), and researchers.

### Digital Medical Platforms

Two of the most popular digital medical platforms, *haodaifu* (“The Good Doctor”) and *zuoshouyisheng* (“The Left-handed Doctor”), in China were not recommended in the selected studies. These two platforms, among others, should proactively be advocated as effective mental health interventions for undergraduate students, especially given the widely acknowledged stigma attached to mental health concerns [50]. In saving undergraduate students’ face and protecting their privacy, digital medical platforms should be popularized among undergraduate students and the general public. Internet-based interventions have been confirmed to be effective for treating common mental disorders [44-46].

### Conversational Agents

The chatbot, as the most popular type of conversational agent, simulates human conversations to provide medical and health care interventions. These human-like, empathetic chatbots are capable of monitoring people’s health [105]. Chatbots and conversational agents display many advantages unmatched by other health consultation alternatives, such as easing the overburdened contact centers and decreasing health risks caused through personal contact [106], providing the only possible solution to catering to the unprecedented demand for health-related information given the lack of professional human agents [107,108], providing timely services at any time [109], ensuring consistent quality services [110], and avoiding moral judgment of user information [111]. Chatbots need to be recommended as an effective mental health intervention tool to undergraduate students, who can try an app on *zuoshouyisheng* that is equipped with the chatbot function.

### Researchers

Only two of the included studies mentioned the role of researchers [70,85]. In fact, researchers can effectively intervene in undergraduate students’ mental health by synthesizing interventions proposed in previous studies and by reporting novel intervention strategies. These synthesized and novel interventions can inform various stakeholders, including educational authorities, college and university management, students’ affairs counselors, and mental health providers. Although playing a role of indirect intervention, their part should never be overlooked.

### Comparison With Previous Work

In this review, we synthesized the interventions for addressing Chinese undergraduates’ mental health concerns into two categories of social support and coping strategies, confirming the findings of these two effective protective factors for mental health highlighted in previous studies [100-103]. Social support from family members, friends, colleagues, relatives, and neighbors [104], and from educational authorities, college and university management, students’ affairs counselors, and mental health providers proposed in many of the 49 included studies can deliver critical and effective interventions, mitigating mental health risks imposed on college students [28-30], sustaining them against crises and stress [32], moderating the relationship between stress and depression [33], and buffering mental health concerns [34]. Previous studies showed that younger adults and people with greater social strain but less social support suffer worse mental health, and that perceived social support impacts the overall depression outcome and the recovery from affective disorders [104,112-114]. The importance of various forms of social support can never be overemphasized in the interventions in Chinese undergraduates’ mental health concerns.

Eight of the 49 included studies reported positive coping strategies [52,82,83,85,86,89,92,93], which effectively improved undergraduates’ mental health conditions. Four of the 49 publications proposed negative coping strategies [52,59,74,75,89], which may cause disastrous consequences. Coping strategies refer to the thoughts and behaviors used by individuals to manage the internal and external demands of stressful events [115]. Although various forms of social support turned out to play an essential role in mitigating Chinese undergraduates’ mental health concerns, these stakeholders’ own coping strategies are more essential. When people face challenging or intricate negative events, the coping style they adopt, be it positive or negative, is crucially important, which will influence their psychosocial outcomes and especially their mental health [115]. People adopting positive coping strategies were afflicted with less emotional distress, whereas those adopting negative coping strategies suffered more emotional distress [116].

Eleven of the 49 included studies described evidence-based internet-delivered digital mental health interventions such as internet-based cognitive behavioral and mindfulness training programs, online brief interventions, online sacramental ceremonies, internet-delivered cognitive behavioral therapy, and others [26,53,55,56,60,71,73,75,77,80,81]. These reported internet- or technology-based interventions can easily be incorporated into existing mental health service provision portfolios. The internet is likely to be an ideal channel to provide promising interventions for students in tertiary education [43,117]. Web-based depression and anxiety interventions were proven effective in treating common mental disorders [45,46]. An internet-based intervention complex, comprising a mental health literacy/destigmatization intervention, a feedback intervention, and a help-seeking list intervention, was proposed by Mowbray et al [44] to promote mental health help-seeking, improve mental health–related knowledge, and decrease the stigma attached to mental health concerns. Online interventions have the potential to fulfill a function in decreasing the depression and anxiety prevalence in the target populations [44]. According to a very recent Canadian study, organizations have been expanding the use of virtual care and digital mental health interventions such as web-based programs, apps, and websites [118]. This is a prevalent trend worldwide. The rapid shift from the use of traditional interventions to the use of digital mental health services and interventions [119] can inform stakeholders of the necessity to adopt digital interventions to support undergraduates’ mental health.

The quality and accessibility of mental health can be improved through mobile apps [120]. Chatbots are one of the main mobile apps for mental health [121]. Chatbots were used to deliver psychological service for medical professionals and the public in China [122,123]. However, none of the 49 included studies proposed chatbot-delivered interventions in undergraduates’ mental health issues. Chatbots have been pervasively used in the field of mental health [121], contributing to addressing the shortage of mental health care [124]. They promise to be an ideal tool that delivers interventions to people suffering from mental health concerns, especially those reluctant to seek mental health advice due to stigmatization. Stakeholders concerned about Chinese undergraduates’ mental health should adopt and popularize this new technology.

### Limitations

This review had several limitations. First, we merely searched two databases (PubMed and ProQuest) for eligible publications. Therefore, there are possibly articles left uncovered in this review. In further research, we will consider more databases, including Embase, CINAHL, PsycInfo, ACM Digital Library, and others. Second, some principal findings may have a low generalizability, considering that most interventions were only reported in only one or two selected articles. Third, we did not compare the findings of this review with other systematic reviews, as this review was the first of its kind.

### Conclusions

Considering that colleges and universities in China have reported unprecedented numbers of students in psychological distress in recent years [2], we performed the first systematic review of interventions addressing the mental health concerns of Chinese undergraduate students studying in China. We found that it is necessary to review this topic systematically, considering the deteriorating mental health of Chinese undergraduate students, especially in the context of COVID-19 resurgences. We divided all the interventions reported in the selected studies into two categories: (1) social support from government authorities, university authorities, students’ affairs counselors and teachers, family members, health care authorities and professionals, and the media (various online platforms), and (2) various coping strategies adopted by undergraduate students themselves. We identified further research on mental health interventions through digital medical platforms, conversational agents (eg, chatbots), and researchers. These interventions combined can provide important implications for practical interventions in the mental health concerns of college students. The intervention in undergraduate students’ mental health concerns is a research topic worth further investigation.

## Appendix

Multimedia Appendix 1 Characteristics of the eligible studies.

## Abbreviations

PRISMA: Preferred Reporting Items for Systematic Reviews and Meta-Analyses
