# Supplementary material for: Interventions in Chinese Undergraduate Students’ Mental Health: Systematic Review
Source: Interact J Med Res. 2022 Jun 15;11(1):e38249. doi: 10.2196/38249 (PMC9244660; doi:10.2196/38249)
Supplement: Multimedia Appendix 1 [file ijmr_v11i1e38249_app1.docx]

Multimedia Appendix 1. Characteristics of the eligible studies (Table S1).

| Reference Number | Authors | Year of Publication | Country | Target Population (Participants) | Study Design/Method | Research Aim | Mental Health Disorder Type (e.g.,depression, anxiety, etc.) | Interventions | Study Limitations |
| --- | --- | --- | --- | --- | --- | --- | --- | --- | --- |
| 2 | Yiman Huang; Xiaoyou Su; Mingyu Si; Weijun Xiao; Hao Wang; Wenjun Wang; Xiaofen Gu; Li Ma; Jing Li; Shaokai Zhang; Zefang Ren; Youlin Qiao | 2021 | China | Chinese undergraduate students | A cross-sectional study through distributing an online  questionnaire | to explore the effects of coping style and perceived social support on the psychological well-being of  college students and relevant risk factors | Depression, anxiety and stress | • Active coping strategies helped improve their psychological well-being.  • Family support was particularly important for maintaining their mental health and ameliorating mental health challenges in this major health crisis.  • Suitable psycho-intervention, routine screening for risk behaviors, and provision of further social support are needed for undergraduate students in the COVID-19 pandemic or other emergency public health events. | 1. The convenient sampling method and the nature of the survey (internet-based) limit the generalizability of the survey results by generating selection bias. 2. All the measurements in the current study were assessed by self-report; thus, response bias may exist. 3. Females and medical students comprised the majority of the sample in this study, and these two groups may be at additional risk for psychological distress during the COVID-19 pandemic. |
| 12 | Xiaosheng Lei; Chaojie Liu; Heng Jiang | 2021 | China | Chinese college students | A cross-sectional study through distributing an online questionnaire | to determine the mental health status and its associated factors among college students in China | Mental health disorders (e.g. obsessive,  interpersonal sensitivity, depression, etc.) | • Universities should develop a care culture and environment that supports the life adjustment of college students, promotes cultural and sports activities, and facilitates the expansion of social networks.  • Mental health education and psychological counselling services should be strengthened. These include hotlines offering timely help to these in urgent needs.  • Early detection and effective management of mental health problems can effectively reduce serious mental health disorders. | 1. The study sample was drawn from one university. Since mental health is highly context dependent, the results can not be extrapolated to the entire university sector. 2. This is a cross-sectional study which could not reflect the development of mental health problems in students over university life. 3. The mental health problems were self-rated by students which may have a recall bias. |
| 26 | Winnie WS Mak; Floria HN Chio; Amy TY Chan; Wacy WS Lui; Ellery KY Wu | 2017 | China | College students and  young working adults | A 2-arm, unblinded, randomized controlled trial | To examine the efficacy of an Internet-based mindfulness training program (iMIND) in  comparison with the well-established Internet-based cognitive-behavioral training program (iCBT) in promoting mental health  among college students and young working adults | Mental health | • It is effective to use Internet-based cognitive-behavioral and mindfulness training programs, which can be easily incorporated into existing service provision portfolios that promote mental health and reduce psychological distress, to promote mental health among college students and young working adults. | 1. The target population was college students and young working adults. 2. The attrition rate of the study is high. 3. A waitlist control group was not included in this study. |
| 48 | Zhiyuan Yu; Lin Wang; Wenyi Chen; Nancy Perrin; Deborah Gross | 2021 | China | Chinese Health science students | A cross-sectional study through distributing an online  questionnaire | to examine the prevalence of adverse childhood  experiences (ACEs) among health science students in China; associations between the  number of ACE exposures and severity of depressive and anxiety symptoms; and the  extent to which resilience moderates the effect of ACEs on mental health outcomes. | depression and anxiety | • Screening for ACEs and strength-based, trauma-informed interventions on fostering resilience is needed to promote mental well-being among Chinese young adults. | 1. this study used a cross-sectional design which limits the inference on causal relationships among ACEs, resilience and mental health outcomes; 2. because all of the data collected was self-reported, results may be susceptible to shared method bias (Podsakoff et al., 2012) and recall bias; 3. this study included a voluntary sample of health science students recruited from one university, a socioeconomically privileged group of young adults; 4. the ACE measure used in this study did not capture several common childhood adversities that may be specific to Chinese sociocultural context, such as parental absence due to internal migration and corporal punishment in schools (Zhang et al., 2020). |
| 49 | Wenpei Zhang; Shankuo Xiong; Yelianghui Zheng; Jinna Wu | 2022 | China | Chinese college students | A cross-sectional study through distributing an online  questionnaire | to explore  the impact of perceived threat on psychic anxiety among college students in the early stage of the COVID-19 pandemic and the mediating roles of response efficacy and self-efficacy | Psychic anxiety | • In practical interventions, authorities (e.g., governments and universities) should first focus on improving efficacy appraisal by providing psychological support to gain the trust of college students so that they believe in and comply with scientific prevention and control measures. By inviting psychiatrists to deliver lectures, authorities can reasonably and effectively enhance the public information of COVID-19-related knowledge and scientific prevention and control measures. | 1. the study was conducted based on cross-sectional data, making it difficult to confirm the causal relationships between variables; 2. the study used self-reported data, which may be affected by a social response bias; 3. many confounding factors are associated with the study variables, such as personality and attitudes; however, this study only controlled for sex, physical health status, and level of risk in participants’ current living area. |
| 50 | Yanmei Shen;Yaru Zhang; Bella Siu Man Chan; Fanchao Meng; Tingyu Yang; Xuerong Luo;  Chunxiang Huang | 2020 | China | Chinese medical college students | A cross-sectional study through distributing an online  questionnaire | to examine the prevalence of anxiety and the associated risk factor of anxiety disorder as well as to explore the association between Attention Deficit  Hyperactivity Disorder (ADHD) symptoms,  depression, suicidal behaviors and anxiety | Anxiety | • It is important to address ADHD symptoms among students with anxiety.  • It is of importance to screen medical students for anxiety disorders in order to better promote the mental health and well-being of this population and better prevent suicidal behaviors. | 1. As a large proportion of the participants are females, it might not represent all medical students in China. 2. As the measurement tool used in this study was self-reported assessment scales, a bias may have resulted from participants’ stigma towards mental health conditions. 3. An absence of priori analysis is a limitation, so in future, researchers should conduct a priori analysis before conducting studies similar to the current study. 4. As a cross-sectional study was employed for this study, its findings were insufficient to draw any conclusions about causal relationships between anxiety and other variables. |
| 51 | Katherine Y. P. Sze; Eric K. P. Lee; Rufina H. W. Chan; Jean H. Kim | 2021 | China | Urban Chinese university students | A cross-sectional study through distributing an online  questionnaire | To examine the prevalence and factors associated with EE among urban Chinese university students | Depression and stress | • Given its associations with negative emotions and other aspects of health, screening and management of EE may improve multiple areas of health and well-being. | 1. Due to the cross-sectional study design, the direction of many of the significant associations (e.g. negative EE and depression) cannot be ascertained conclusively. 2. Students from these two universities may not be fully representative of all tertiary institute students in Hong Kong. 3. Only nine male participants engaged in negative EE and this could limit the power to detect the association between EE and psycho-social factors among male students. 4. There were potential confounding variables that were not covered in this study such as social support and the presence of eating disorder psychopathology. |
| 52 | Nan Zhao; Guangyu Zhou | 2020 | China | Chinese college students | A cross-sectional study through distributing an online  questionnaire | To investigate associations of COVID-19-related  social media use with mental health outcomes and to uncover potential mechanisms underlying the links | Depression | • It is critical for policymakers, public health agencies, parents, psychologists, and health-care staff to remain sensitive to the potential negative consequences of ubiquitous social media exposure.  • The general public, especially those who have been directly or indirectly traumatized by COVID-19, could be advised to avoid excessive social media use and learn effective emotion regulation strategies (e.g. reappraisal) to reduce negative emotions induced by news coverage. | 1. Participants were fully recruited via social media and those who did not use any type of social media may not be included in the study, posing a potential threat to representativeness of the sample. 2. The time lag between the measurement of social media use and negative affect is not short enough to assess the immediate responses to disaster messages. |
| 53 | Mingli Yu, Fangqiong Tian, Qi Cui and Hui Wu | 2021 | China | Chinese college students | A cross-sectional study through distributing an online  questionnaire | to investigate the prevalence and the  related factors of depressive symptoms among Chinese college students | Depression | • The government can open free psychological hotline consultations to help college students solve their  psychological problems.  • The media should release correct information timely and prevent the spread of rumors.  • Universities can actively organize health education activities and encourage college students to arrange their time reasonably and take the initiative to find a suitable way to relieve stress during home quarantine. | 1. this cross-sectional study cannot allow us to demonstrate the causal relationship between variables 2. self-reported questionnaire was conducted online, which will lead to recall bias and response bias to some extent 3. in addition to the vari-ables we took into account, there may be other factors that were associated with the prevalence of depressive   symptoms in college students |
| 54 | Yue Li and Jun Peng | 2021 | China | Chinese college students | A cross-sectional study through using the coping strategy questionnaire, social support questionnaire, and self-rating anxiety scale | to examine the relationships between coping strategies, social support, and  Anxiety and to delve into the potential mediating effect of social support on these relationships | anxiety | • Adopting positive coping strategies may enhance social support that in turn relieves anxiety. The effect of social support, especially family and counselor support, can decrease anxiety in coping with the COVID-19 pandemic cognitively and behaviorally.  • Policymakers and school administrators should encourage meaningful communication between family members and activate effective counseling services to maintain positive mental health. | 1.the samples were restricted to one university, which may be less generalizable to the entire population  2.the cross-sectional design of this study could not reflect the long-term development of the samples, which needed supplementary findings from longitudinal research  3.all the questionnaires were self-reported, and the veracity of the result relied on the self-perception of the subjective and non-subjective methods should be applied  4.anxiety as a dependent variable involved in the survey could not reflect an intact picture of mental health, and it is  necessary to further explore other factors indicating traumatization, such as depression, fear, despair, and irritability |
| 55 | Tingxuan Wang, MPhil; Janet Y H Wong, PhD; Man Ping Wang, PhD; Amanda Chiu Yin Li; Sang Suk Kim,  PhD; Jung Jae Lee, PhD | 2021 | China | Chinese college students | Structural Equation  Modeling Approach Using a Cross-sectional Online Survey | to test the mediating role of SNS addiction between SNS use patterns and mental health status  among Chinese university students in Hong Kong (HK) | Mental health | • reducing SNS addiction and mental problems by conducting interventions using cognitive-behavioral  approaches.  • Screening for and addressing excessive SNS use are needed to prevent SNS addiction and mental distress  among young people. | 1.Cross-sectional data in this  study could not provide the causality between SNS addiction and mental health status  2.Discussions of the magnitude of the  relationship between 2 elements (SNS addiction and mental health status) were inconclusive  3.as most of our participants were ethnic Chinese in HK, replication of our study for young adults in other sociocultural settings is recommended to reveal a more  comprehensive relationship that was discovered through this study  4.the participants responded to an online survey.  Self-reported data may result in recall bias. |
| 56 | Hao Fong Sit, Ieng Wai Hong, Sebastian Burchert , Elvo Kuai Long Sou, Mek Wong,  Wen Chen, Agnes Iok Fong Lam and Brian J. Hal | 2021 | China | Chinese college students | An uncontrolled, feasibility trial | To address depression and anxiety symptoms in Chinese young adults,to assess feasibility of recruitment  and of delivery of Step-by-Step in a University setting, to assess acceptability of the  intervention, and to examine potential effectiveness | Depression | • evidence-based digital mental health interventions | 1.Although a feasibility or pilot study is common practice and recommended for evaluation of recruitment potential, preliminary effect size and sample size estimations, and safety assessment before running the main trial, the results should be cautiously interpreted , and the effect sizes observed among completers may not be observed in the full trial  2.The current study design was an uncontrolled, single-arm pilot trial with a small sample  size and not fully powered to detect significant changes over time  3.the current study piloted SbS in one residential college in one  University, which limits generalizability  4.Our results revealed that a larger proportion of the participants were female, which might limit the interpretation of the results of this population |
| 57 | Shun-Wei Liang, Rong-Ning Chen,Li-Li Liu, Xue-Guo Li, Jian-Bin Chen,Si-Yao Tang and Jing-Bo Zhao | 2020 | China | Guangdong  college students | Fear Screening Scale, Patient Health Questionnaire, and Impact  of Event Scale-6 | To investigate  the relationship between college students’ mental health status and psychological help-  seeking behavior to test the phases-decision-making model (PDM). | Fear, depression, and trauma | • University campuses should develop and implement effective screening procedures to closely monitor students’ exposure to stressors and mental health status.  • We should design a psychological intervention program for fear and fully utilize psychological assistance hotlines to help college students better adjust themselves.  • performing psychological help-seeking intervention, strengthening the dissemination of mental health knowledge, and improving the level of mental health perception are effective ways to improve help-seeing attitudes and increase the probability that college students will seek psychological help. | 1.the cross-sectional design could not explain the cause–eﬀect relationships and does not allow the investigation of changes in individuals’ mental health status and psychological help-seeking behavior across diﬀerent periods of the COVID-19 epidemic  2. because all the constructs were assessed by self-report, the estimated relations among fear, depression, trauma, and psychological help-seeking behaviors might be subject to response bias  3.due to the use of the convenience sampling method, the study sample primarily comprised college students in Guangdong Province.  Future research needs to expand the scope of the survey to other provinces and cities in China and carry out stratiﬁed sampling to obtain a more comprehensive understanding of the situation of Chinese college students  4.this survey lacked the measurement of the three processes of seeking psychological help, namely, help-seeking attitudes, intentions, and behaviors,limiting the explanatory power of the results |
| 58 | Mohammad Nurunnabi , Syed Far Abid Hossain Hossain et al. | 2020 | China | Chinese college students | an online survey using a semi-structured  questionnaire with a simple random sampling technique | to discover the coping strategies of Chinese students for anxiety during the  COVID-19 pandemic in China. | anxiety | • The university authority should be aware of the students’ coping strategies. In particular, students who live without parents or relatives should be taken care of properly during the outbreak.  • To help students cope with the mental pressure, university authorities may think about arranging or organizing programs such as an online experience-sharing competition, and encourage students by offering rewards or financial aids.  • Required food and healthcare materials should be supplied to ensure the students’ safety. | 1.the study is limited  to students in China from different universities and majors  2.the respondents are selected randomly due to online data  collection from various provinces that were not affected equally.findings should not be generalized to the overall student group  3.the most affected and least affected  areas should be separately investigated to fully analyze the phenomenon   1. This research is based on empirical analysis, which may affect the generalizability of the research   5.if the university authority, administrative staffs and teachers could  be selected as respondents, the study might produce more interesting findings. |
| 59 | Ruipeng Wu  , Lan Guo et al. | 2021 | China | Chinese college students | an online survey that included Eating Attitude Test-26，the Chinese Version  of Pittsburgh Sleep Quality Index (CPSQI), Smartphone Addiction Scale—Short Version  (SAS-SV)，Patient Health Questionnaire-9 (PHQ-9) and  Generalized Anxiety Disorder-7 (GAD-7) | to examine potential serial multiple  mediation effects of problematic smartphone use (PSU) and psychological distress  (i.e., depressive and anxiety symptoms) in the relationship between sleep quality and  disordered eating behaviors/attitudes (DEBs) | Psychological Distress | • Sleep hygiene, mobile phone and internet use hygiene, mental health education courses, professional psychological counseling and other interventions should be considered and implemented.  • appropriated interventions that target problematic smartphone use could potentially reduce anxiety and depression level, which will in turn provide a buﬀer against the negative impact of poor sleep quality on eating disorder symptoms. | 1.the current study proposes a preliminary exploration for the associations, where longitudinal studies are greatly needed to  further assess the causal relationship  2.the data was collected by self-reported measures, so reporting bias may  be introduced. Nonetheless, self-reported questionnaires were proven to be valid and applied worldwide  3.although a number of potential confounders were included, there are some  unmeasured confounders (e.g., parenting styles, substance use and other variables) that may contribute to these associations  4.the current study only includes college  students who are currently on campus and did not account for those absent |
| 60 | Xu Chen  , Xiaolong Zhang  , Xuequan Zhu  and Gang Wang | 2021 | China | Chinese college students | a randomized  controlled trial | to evaluate the efﬁcacy of MoodBox, an online psychological intervention  program, for subclinical depression | Subclinical depression | • an Internet-Based Intervention for Subclinical Depression (MoodBox) that is a web-based psychological intervention informed by evidence-based psychological interventions, including CBT, IPT, and mindfulness meditation. | 1. all participants will be recruited from ﬁrst-year university students in Beijing;  thus, the sample might not be representative of the subclinical depression population in China  2.although the length of treatment and follow-  up period (i.e., after the 8-week intervention, participants will be followed up for 1 year) is suﬃcient to demonstrate treatment eﬀect, due to the relatively short follow-up period, the long-term eﬀect of the intervention cannot be evaluated in this study. |
| 61 | Yanqiu Yu1, PhD; Rui She et al. | 2021 | China | Chinese college students | A cross-sectional study through distributing an online  questionnaire | To investigates the levels of depression and mental distress due to COVID-19, and the associations between  cognitive, behavioral, and psychosocial factors, and depression and mental distress due to COVID-19 among university students  in China. | Depression and Mental Distress | • Various cognitive, behavioral, and psychosocial responses to COVID-19 showed both direct and indirect effects (via mental distress due to COVID-19) on depression. Thus, interventions to improve such multidimensional factors might reduce mental distress during the initial COVID-19 outbreak period. | 1.it did not have national coverage. Selection bias may exist, as classes and departments were not randomly selected  2.we did not cover important interpersonal factors (eg, subjective norms and social support), which were associated with many health-related behaviors  3.the relatively mild magnitudes of some mediation and suppression effects of mental distress due to COVID-19 imply existence of other unstudied mechanisms  4.the cross-sectional study design does not allow for causal inferences,as depression may also change perceptions |
| 62 | Zhang, Yueyang;Zhao, Jingjing;Juzhe Xi;Fan, Bingbing;Wang, Qiong;Yao, Zhiying;Huang, Tianhui;Bai, Han | 2021 | China | 4520 undergraduates in different disciplines from five Chinese universities | An online questionnaire was used for the survey, and the PTSD questionnaire (PCL-C) was used as a screening tool | This study researched the prevalence and associated factors of home posttraumatic stress disorder (PTSD) among Chinese college students during the covid-19 pandemic. | post-traumatic stress disorder (PTSD) | • The paper recommends providing relevant education and psychological counseling to parents during the outbreak to help them understand their children's mental state, and suggests that universities provide psychological counseling and psychological interventions to students, focusing on college students who are most severely affected by the epidemic. | 1. The study did not measure some other variables that might affect the outcome 2. The online questionnaire yielded data based on self-assessed data, which was highly subjective and may have recall bias 3. The paper covers college students, and the results may not be applicable to other populations, such as adults or the elderly. 4. The use of a cross-sectional design cannot provide strong evidence of causality. |
| 63 | Li, Na; Fan, Lurong; Wang, Yan; Wang, Jing; Huang, Yu | 2022 | China | 6,027 Chinese university students | a cross-sectional study | This paper aims to evaluate the impact of the Covid-19 pandemic on psychological distress and risk factors for psychological disorders, including coping style and emotional regulation | different levels of PD and psychological disorders | • The results of this study could help healthcare professionals identify college students at high risk of mental health problems so that appropriate interventions can be targeted against them.The study recommends providing long-term psychological services for students. | 1. This is the first time to assess the relationship between coping styles, emotion regulation, and PD, and therefore, replication is necessary to confirm our findings. 2. This study only examined coping styles and emotion regulation strategies.Subsequent studies should involve more psychometric measures. 3. Subjects were limited to Chinese college students from one school, and later studies should be extended to all occupational groups and ethnic samples 4. The study interviewed younger subjects with a younger age range with limited benefit and should then be studied in a larger age range thereafter |
| 64 | Tang, Wanjie;Hu, Tao;Hu, Baodi;Jin, Chunhan;Wang, Gang;Xie, Chao;Chen, Sen;Xu, Jiuping | 2020 | China | in a sample of homequarantined Chinese university students | Questionnaires were used, and data were collected on sleep durations, exposure, home-quarantine time and socio-demographic variables. | This study researched the risk factors of psychological distress from the prevalence of posttraumatic stress disorder (PTSD) and depression among home-quarantined college students. | PTSD and depression | • The paper recommends providing psychological interventions for quarantined college students to help them reduce fear and improve sleep duration.Universities need to consider planning acute and long-term psychological services for more vulnerable students, graduates, and students living in the most severely affected areas. | 1. The paper studied college students and the findings may not be applicable to other adults or the general population. 2. The study did not measure some other variables that may have affected the findings. 3. Online self-assessment and non-rigorous random sampling may reduce the representativeness and reliability of the results. |
| 66 | Zhou, Wenjing;Li, Peizhi;Lei, Xu;Yuan, Hong | 2021 | China | Southwest University undergraduate and graduate students | Control experiments were used | The study aims to evaluate the effects and impact of positive mental imagery training on Chinese students with depressive symptoms | Depression,  negative emotions | • A week of positive mental imagery training can help to improve negative emotions and anxiety in depression.The paper suggests a further exploration of this training program. | The study had a potential high risk of bias and weak control conditions |
| 66 | Shen, Yanmei;Bella Siu Man Chan;Huang, Chunxiang;Cui, Xilong;Liu, Jianbo;Lu, Jianping;Patel, Marguerite;Verrico, Christopher D;Luo, Xuerong;Xiang Yang Zhang | 2021 | China | 5,693 Chinese medical college students | a cross-sectional study | The study explored the prevalence of suicidal behaviors, and its association with ADHD among Chinese medical students. | Suicidal behaviors and attention deficit hyperactivity disorder (ADHD) | • Providing mental health care and counseling services to students at high-risk groups in medical schools.  • The early diagnosis and treatment of ADHD may have a suicide prevention effect. | 1. Suicide data were derived from participant self-reports, which may have implications for the validity of the results and may have a recall bias. 2. The scales used in the study may be biased in reporting the symptoms. 3. The study did not consider some unknown mixed factors that may modulate the relationship between suicidal behavior and ADHD. 4. The participants of the study were medical students, and the findings could not be generalizable to the entire population. 5. Participants were mainly female, with small numbers in the ADHD-H subgroup, which may create a selection bias. 6. The sample size of ADHD-H subtypes is small and support for the conclusions may be insufficient. |
| 67 | Li, Dandan;Zou, Li;Zhang, Zeyu;Zhang, Pu;Zhang, Jun;Fu, Wenning;Mao, Jing;Cao, Shiyi | 2021 | China | Home-Quarantined Nursing Students in China | a cross-sectional study.  Online questionnaire survey,  Use GAD-7,PHQ-9,PCL-C for the evaluation and Logistic regression analyses to determine the risk factors. | The purpose of the study was to determine the prevalence and major determinants of anxiety, depression, and post-traumatic stress symptoms (PTSS) among Chinese nursing college students during the isolation of the covid-19 pandemic | anxiety, depression and post-traumatic stress symptoms  (PTSS) | • Parents strengthen communication with their children and provide psychological support to their children.  • Universities carry out relevant online mental health courses and implement psychological intervention measures to improve students' psychological adaptability. | 1. The study could not determine the causal relationship between anxiety, depression, PTSS, and variables. 2. Self-assessment scales may cause a response bias in the results. 3. The subjects were nursing students, and the findings could not be extended to students in other majors. 4. The study did not investigate the impact of knowledge and behavior about the Covid-19 pandemic on psychology. 5. The scales used in the study did not pilot test nursing students to assess the face validity of these scales. |
| 68 | Kai‐Han Yang;Wang, Lei;Liu, Hui;Lin‐Xia Li;Xiao‐Lian Jiang | 2021 | China | university students in Sichuan Province, China | An online cross-sectional study | This study aims to evaluate the effect of COVID-19 on the mental health of college students in Sichuan Province, China. | mental health | • The government, school administrators and society to strengthen operability research to provide coping strategies and to implement psychological interventions and conduct relevant training. | 1. The study was only conducted in Sichuan Province, China, which may not generalize the results to other regions of China or other countries. 2. The analysis of risk factors is based on only a few simple factors, which may reflect inaccurate mental health outcomes. 3. The psychological state of college students may not only be caused by covid-19, but also because other psychological problems in themselves were aggravated during the epidemic. |
| 69 | Liu, Kunyan;Duan, Yunfei;Wang, Yilin | 2021 | China | 1,000 second-year undergraduate students at Harbin  University of Science and Technology, China | with the short form of the Positive and Negative Affect Schedule (PANAS) | This paper aims to study the effectiveness of online positive psychology  intervention (PPI) to improve students' mental health status | mental health | • Universities should adopt a web-based positive psychology intervention (PPI) to improve the mental health of college students. | 1. The study lacked the specific demographic information. 2. The paper used measures not designed for medical conditions, such as clinically diagnosed depression or anxiety disorder; therefore, it is unclear whether web-based PPI has a significant impact on the resolution of mental illness symptoms. 3. There were temporal differences between the experimental and control groups, which may contribute to the risk of increased psychological stress in both groups |
| 70 | Carciofo, Richard | 2020 | China | 625 university students from Xi’an Jiaotong–  Liverpool University, Suzhou, China | Online questionnaire survey | Morning affect, eveningness, and amplitude distinctness: associations with negative emotionality, including the mediating roles of sleep quality, personality, and metacognitive beliefs | Depression, anxiety, and stress | • Longitudinal studies of these variables may establish causal relationships and may inform interventions to treat psychological distress and disorders. | 1. The sample size was small may lead to the results were not universal. 2. Different questionnaire scales may lead to different outcomes. |
| 71 | Yen, Ju-Yu;Yen, Cheng-Fang;Wu, Hsiu-Yi;Huang, Chun-Jen;Ko, Chih-Hung | 2011 | China | college students from Taiwan | the Chen Internet Addiction Scale, the Center for Epidemiological Studies’ Depression  Scale, and the questionnaire for online activity. | The paper examines whether the difference  between hostility in the real world and networks are related to Internet addiction, depression, and online activity | Depression | • The study found that depressed college students have less hostility after entering the Internet, suggesting that the Internet become one of the media to provide treatment for people with depression. | 1. The cross-sectional study design of this study could not confirm a causal relationship between Internet use and hostility. 2. Assessment of internet addiction and depression was based only on self-reported information from college students. |
| 72 | Zheng, Xiaolei;Guo, Yuji;Ma, Wen;Yang, Hui;Luo, Liyan;Li, Wen;Zhou, Xiaolan;Li, Qing;Bi, Jianzhong;Wang, Ping;Wang, Hongxing | 2021 | China | 300 Chinese undergraduate students from Jinan City | an online longitudinal survey was conducted via a WeChat applet | This paper aims to explore the differences in mental health problems among college students during the peak and reopening period of the COVID-19 epidemic in China | depression and anxiety | • The paper recommends adequate social support and long-term targeted psychological intervention for college students.  • The study indicates more serious mental health problems among fourth-grade students and proposes to specifically increase their employment opportunities and develop mental health rehabilitation programs. | 1. The psychological assessment used in the study was based on a less informative online survey and self-report scale, which may have selection bias. 2. The psychological problem assessment was not comprehensive enough to consider some other factors that may lead to the occurrence of depression and anxiety in college students. 3. The small participation number of the study made it cannot prove that the participants represented college students from all universities in Jinan. |
| 73 | Bijia Song, Yilin Zhao,  , Junchao Zhu | 2020 | China | Native Chinese students attending university in the U.S | Chinese Star Survey website | to survey international students to better understand their traumatic effects and psychological  reactions from COVID-19, to develop evidence-driven strategies to reduce adverse psychological impact during the pandemic. | Depression, Anxiety, and Stress | • online or smartphone-based psycho-education and psychological interventions that will also reduce the risk of virus transmission by forgoing face-to-face therapy. | (i)the study focused on Chinese international students, the results may not apply to other adults or the general population.   1. personality and coping styles of different participants, which may influence the results, were not measured in this study. 2. our research only represents the mental status of students in this particular period. 3. we did not adjust for baseline depression as a covariate. The present sample is also small and was limited to a convenience sample. And the snowball sampling method is susceptible to sampling bias. Since participants are chosen by individuals who have already been selected, it is possible that all of the participants will share certain characteristics or traits. 4. socioeconomic status varies over time in this age range, and could not be assessed in the present study |
| 74 | Xiaoyu Tao, Dong Chen, Yawen Fan, Lanxin Zhang, Houqian Shan, Yi Wei, Xi Yu, Tian Zhong, Ling Wang,  Sookja Kim Chung  , Yaqin Yu and Ying Xiao | 2021 | China | college students in Macao | A cross-sectional study through distributing an online questionnaire | to investigate the effect of the  coronavirus disease (COVID-19) pandemic on the mental health condition and  sleep quality of college students in Macao. | mental  health status | • The unsupervised, self-initiated intervention against mental and sleep disorders of students can lead to more disastrous outcomes. | (i) it was a cross-sectional survey, which  could not explain the cause-and-effect relationship between the COVID-19 pandemic and mental health status or sleep quality.  (ii) Then, the study population was not a random sample and might not avoid bias in subject selection.  (iii) the questionnaire was finished in the form of a self-report; therefore, some of the answers might be subjective. |
| 75 | Yuehui Jia,  Yanbo Qi,  Li Bai,  Yunfeng Han,  Zhiping Xie,  Jie Ge | 2021 | China | Chinese college students | A cross-sectional study through distributing an online questionnaire | to investigate the  knowledge–attitude–practice (KAP) of Chinese college  students regarding COVID-19 and evaluate their  psychological status against the background of the  COVID-19 outbreak. | anxiety | • Public health education from health authorities in various governments is needed for the dissemination of the importance of preventive measures during COVID-19.  • Psychological health services should be implemented to alleviate the adverse effects of this pandemic under national social distancing. Psychological interventions could also be carried out through online platforms under national social distancing during COVID-19.  • Teachers should also pay attention to strengthening the dissemination of COVID-19 knowledge and preventive measures to reduce the level of anxiety and depression in the student group. | (i)The online survey could not control the sample size of the study as the subjects voluntarily participated.   1. The sample was not randomly selected, thus, a possible selection bias could not be ruled out. |
| 76 | Yaoshan Dun, Jeffrey W. Ripley-Gonzalez,  Nanjiang Zhou,  Baiyang You,  Qiuxia Li,  Hui Li,  Wenliang Zhang,  Randal J. Thomas,  Thomas P. Olson,  Jie Liu  Yuchen Dong,  Suixin Liu | 2021 | China | Chinese college students | A retrospective observational study | To observe the weight change in Chinese  youth during a 4-month COVID-19 lockdown, and the  association between weight change and mental health,  physical activity and sedentary time changes, and dietary  habits. | stress, depression,  anxiety | • Interventions to decrease sedentary time and improve mental health may be warranted to mitigate weight gain during the lockdown period and reverse the weight gain in youth after the COVID-19 pandemic. | Not Given |
| 77 | Jincong Yu  , Ziyun Yang  , Yuqin Wu  , Ming Ge  , Xuemei Tang and Hongbo Jiang | 2021 | China | Chinese college students | web-based survey and questionnaire | to understand the prevalence of and factors associated with depressive symptoms  after a long quarantine time and online learning at home among college students in  Wuhan, China. | depression | • Grief counseling and online sacramental ceremonies should be implemented for this group to prevent negative emotional difficulties.  • mindfulness meditation and cognitive behavioral therapy, to reduce student’s anxiety and depression. | 1. the cross-sectional design complicates causal inference. 2. a nonrandom sampling method was used, and the students were from the same college in Wuhan; therefore, the sample was not fully representative of all Chinese college students although these students were from all regions of China. 3. the status of depressive symptoms was determined by an online questionnaire rather than a clinical diagnosis. 4. some mediation or moderation effects were not considered in our study. Largescale studies with longitudinal designs and randomized sampling methods as well as clinical diagnoses for depressive symptoms should be conducted in the future. |
| 78 | Jia-Yan Pan1 and Xiaoyu Zhuang | 2021 | China | college students in Hong Kong | online questionnaire | to develop an adventure-based cognitive behavioral intervention (aCBI) program and tests its  effectiveness in improving the mental health of Chinese university students in Hong Kong. | stress | • The integration of cognitive behavioral intervention and adventure training in a class setting might be an effective and feasible approach for the mental health counseling of university students. | 1. this study was not a randomized controlled trial (RCT). There was no control group involved, so future studies should further test its effectiveness with an RCT. 2. This lack of response may cause a non-response bias of the findings. 3. Future studies should consider two classes or more during each semester to allow more students to attend the program. 4. Future studies are advised to recruit both undergraduate and postgraduate students in multiple universities within or beyond Hong Kong to further validate the effectiveness of this aCBI program. |
| 79 | Larry Auyeung   · Phoenix Kit Han Mo | 2018 | China | Chinese college students | 6-day positive psychological intervention (PPI) | to examine the efcacy and mechanism of a 6-day online self-help PPI on improving  eudaimonic well-being (fourishing) and reducing depressive symptoms among Chinese  university students. | depression | • positive psychological intervention (PPI) | 1. three-waves mediation model (i.e., cross-lagged model or a latent growth model) will be a more robust study design to verify this finding. 2. The sample might not be representative of the world population or other rural cities in China; 3. future studies may consider using behavioral or physiological outcome measurement. 4. our analysis was done in a relatively small sample size which barely reached the minimal sample number requirement for modeling. 5. the quality of participants’ refection in the BPS intervention was not monitored. |
| 80 | Chengjia Zhao  Huihui Xu  Xinyi Lai  Xue Yang  Xiaolian Tu  Nani Ding  Yijun Lv  Guohua Zhang | 2021 | China | Chinese college students | the  Perceived Stress Scale, Mobile Phone Addiction Index Scale, Online Social Support  Questionnaire, and Perceived Social Support Scale | to test the relationship between perceived  stress and problematic smartphone usage as well as the mediating role of online social  support and the moderating role of perceived social support among Chinese undergraduates. | Stress | • The mediating effect of online social support was stronger among college students with lower perceived social support than those with higher perceived social support. | 1. we used convenient samples of college students, which will cause sampling bias, and thus the generalization of these findings should be taken seriously. 2. the research depended on a self-report survey. Future research could use a variety of   information providers (eg, peers and parents) and research methods (eg, qualitative interviews) to collect data to better understand individuals’ mental and behavioral status.   1. the cross-sectional design prevents the testing of causal hypotheses. 2. As the subjects in this study are from a university, it may not be conducive to the promotion of our research results. 3. This study failed to identify groups according to SAR (Specific Absorption Rate) values of smartphones. |
| 81 | Meiqi Xin, Sitong Luo, Rui She, and Yanqiu Yu  Lijuan Li  Dali Wang  Le Ma  Xi’an  Fangbiao Tao  Jianxin Zhang  Junfeng Zhao  Liping Li  Dongsheng Hu  Guohua Zhang  Jing Gu  Danhua Lin  Hongmei Wang  Yong Cai  Zhaofen Wang  Hua You  Guoqing Hu  Joseph Tak-fai Lau | 2020 | China | Chinese college students | A cross-sectional study through distributing an online questionnaire | To investigate (a) the associations between mandatory quarantine  status and negative cognitions (perceived discrimination because of COVID-19 and perceived  risk of COVID-19 infection)/mental health status (emotional distress because of COVID-19,  probable depression, and self-harm/suicidal ideation), (b) the associations between the negative cognitions and mental health status, and (c) potential mediations between quarantined status and probable depression and self-harm/suicidal ideation via COVID-19-related  negative cognitions/emotional distress. | emotional distress because of COVID-19,  probable depression, and self-harm/suicidal ideation | • Online brief interventions need to be made available, including screening of mental distress, counseling hot-lines, emotional regulation and coping skills, and promotion of positive psychology | 1. we did not ask about reasons behind perceived discrimination. 2. the cross-sectional design prohibits causal inferences. 3. given the large sample size, associations of small effect sizes could be statistically significant. 4. single items were used to assess perceived discrimination and perceived risk of infection. 5. specific information about the quarantine conditions was not measured (e.g., reasons and venues for the quarantine). 6. the study did not ask about reasons behind the perceived discrimination. 7. generalization of the findings to other populations should be made cautiously, as the sample was nonrandomly selected and limited to university students |
| 82 | Liang, Kaixin, Clarice M. de Lucena Martins, Si-Tong Chen, Cain C.T. Clark, Michael J. Duncan, He Bu, Liuyue Huang, and Xinli Chi. | 2021 | China | 1846 Chinese college students surveyed online in August 2020 | An online questionnaire was used for the survey, and through network analysis and multivariate analysis of covariance | This study aimed to examine the associations between meeting guidelines and mental health among college students. | lifestyle; physical activity; sedentary behavior; sleep; mental health; COVID-19 | • Compared to meeting no guidelines, meeting the sleep guideline (alone or in combination with other guidelines) was associated with significantly lower levels of depression and anxiety; meeting both SB and MVPA guidelines was also associated with a significantly lower level of depression. Hence, meeting more guidelines, especially adhering to a healthy sleep routine, may play an important role in promoting the mental health of young adults. | 1. First, the cross-sectional design precludes confirmation of causality between movement behaviors with depression and anxiety. Physical inactivity, prolonged sitting time, and abnormal sleep duration can also be the consequences of depression or anxiety , or reciprocal associations exist between these movement behaviors and mental health, as some literature has proposed. 2. Second, the self-reported data of movement behaviors and mental health problems, despite PHQ-9 and SAS being psychometrically valid, might have been prone to inaccuracy due to potential recall biases and social desirability. Since accurate assessment of movement behaviors is crucial for defining recommendations for health promotion at a population level, objective measures of movement behaviors, such as pedometers and accelerometers, are preferable in future studies to improve the accuracy of health-related data. Feasible, consumer-grade products(e.g., mobile applications, wearable devices) also make it possible to collect and analyze movement behaviors in a large-scale study. Future research is needed to confirm and build upon this study with objective measures of movement behaviors. 3. Third, our study recruited samples using a convenience sampling procedure, so the representative-Healthcare 2021, 9, 1166 10 of of the study sample cannot be guaranteed. Fourth, the Canadian 24-hour movement guidelines also include a quantitative recommendation on the time spent on recreational screen-based SB (≤3 h), which we did not measure in this study. 4. Finally, although we included a series of confounding variables in our study, some other important correlates were not considered, such as dietary behaviors, which are known to be correlated with mental health problems |
| 83 | Bo Chen, Jinlu Sun, Yi Feng, Yi Fe | 2020 | China | 255 students at a public university in Hong Kong participated in an online-based  survey | an online-based  survey that assessed their perceived available peer support, emotional well-being, and depressive  symptoms. | The present study examined the associations among university students’ perceived available peer  support, emotional well-being (as indicated negatively by loneliness and negative affects and positively  by positive affects and hope), and depressive symptoms. | Social isolation,social distancing， available peer support, emotional well-being, and depression | • Perceived available peer support negatively contributed to depressive symptoms.  • Both negative and positive indicators of emotional well-being mediated the association between perceived available peer support and depressive symptoms and Advanced the practical needs for preventive efforts and accessible care to support the psychological and emotional needs of young people during the COVID-19 pandemic. | 1. The paper studied college students at only one university in Hong Kong,which has a disproportionate female population and the findings may not be applicable to other adults or the general population. 2. Secondly, students themselves were the sole informants in the present study, which may potentially introduce a method bias . Our findings need to be replicated using multiple methods and to collect data from multiple informants. 3. Thirdly, our cross-sectional design and correlational analysis prevented us from making causal inferences 4. 4. Additionally, due to our lack of prepandemic data, we are unable to rule out the possibility that our findings can be explained by students’ earlier levels of depression. |
| 84 | Yin Li, Linbo Qin, Yaobin Shi and Jun Hang | 2021 | China | in a sample of 1168 college students in a university located in Wuhan  during the lockdown | a cross-sectional online survey  was designed to understand the mental state of college students in a university located in Wuhan  city during the lockdown | In this paper, a cross-sectional online survey  was designed to understand the mental state of college students in a university located in Wuhan  city during the lockdown. | severe psychological symptoms such as anxiety, depression, insomnia  and fear to the people isolated at home | • The results of this study showed that above 50% participants had obvious fear and anxiety symptoms; anxiety and fear were 61.64% and 58.39%, respectively.  • Conformity (49.49%), invulnerability (26.11%), insensitivity (21.49%) and rebelliousness (12.41%) symptoms also appeared.  • The senior students experienced more anxiety than the freshmen.  • Psychological symptoms (except for the insensitivity symptom) had no significant difference in gender, residence and annual household income after the one-way analysis of variance. | 1. the study had a limited response of 1168 and all respondents came from  one university located in Wuhan city. The generalizability of this finding was insufficient.  2.this study was undertaken between April and June, which was the medium stage  of COVID-19; the views could not represent the final view such as the influence of infected  relatives on the psychological symptoms of students. A secondary survey was necessary  and the dynamic variations of the psychological symptoms should be tracked.  3.The respondents were not equally distributed with regard to gender, grade and residence.  4.all data from the survey were obtained by self-reporting. |
| 85 | Yuanyuan Zhu, Hongyun Wang and Aihong Wang | 2021 | China | 342 undergraduate nursing students in China during the pandemic. | A Generalized Anxiety Disorder-7 (GAD-7), Patient Health Questionnaire9 (PHQ-9), and Emotion Regulation Questionnaire (ERQ) were used to evaluate mental health  and emotions and using the Statistical Package for Social  Sciences (SPSS) version 22. | The study explores the mental health and emotion regulation  experienced by undergraduate nursing students in China during the pandemic and Potential risk  factors related to negative mental health symptoms | anxiety, depression, or comorbid anxiety and  depression | • This study provide a better understanding of the association between mental health and emotion regulation, which will help direct psychological intervention that relieves these issues during the pandemic. | 1.this study used online social media to  recruit participants, which may have resulted in selection bias. Since most participants were located outside of Hubei province, the representativeness of the population was limited. A follow-up study needs to have an  increase sample size.   1. Signs of mental health illnesses were determined by self-reported analyses instead of professional clinical diagnoses, which can lead to reporting bias. 2. this study used cross-sectional methodology and the causal relationships between mental health symptoms and risk factors could not be established. |
| 86 | Lijun Zhuo , Qian Wu , Hong Le, Hao Li, Ling Zheng , Guoqing Ma and Hongbing Tao | 2021 | China | A total of 1017 participants among back-to-school students in Wuhan | A total of 1017 participants voluntarily  provided sociodemographic characteristics and accomplished the following scales | This study has significant implications to intervene in the mental health of college  students with extreme uncertainty of COVID-19. | intolerance of uncertainty; social support; mental health | • Back-to-school students who are certain and uncertain that COVID-19 will rebound again were significantly more anxious and depressed than those with optimistic attitudes.  • Government departments should pay high attention to the mental health problems evoked by intolerance of uncertainty.  • Social support as a moderator could buffer the relationship between IU and mental health, including anxiety and depression during unprecedentedly uncertain times. | In light of the current literature, no study has explored the moderation of social support between IU and mental health problems among college students during the COVID-19 era. Based on the results above, low COVID-19-related uncertainty and strong social support from friends, teachers, and family members can eliminate mental health problems effectively |
| 87 | Xueyan Li, Ping Fu,Changyu Fan , Miao Zhu and Min L | 2021 | China | 4355 students enrolled in Wuhan universities  and colleges participated | An Online questionnaire survey, and post-Traumatic Stress Disorder via the Impact of Event-Scale-Revised was  assessed | This study aimed to explore influencing factors for the psychological impact of COVID-19 on Wuhan college students, post-traumatic stress symptoms in particular, so as to inform evidence-based strategy development to ameliorate such adverse impacts | close relationship level risks such as family support and online course difficulties as well as distant level risks and post-traumatic stress symptoms  (PTSS) | • Mental health services reducing PTSD should be provided.  • Students who have lost loved ones and suffered family financial loss should be given particular care. | 1. The study was only conducted in Wuhan China, which may not generalize the results to other regions of China or other countries. |
| 88 | Huali Zhan , Chunmei Zheng , Xianqin Zhang , Meng Yang, Lin Zhang and Xu Jia | 2021 | China | A total of 1,586 questionnaire of college students were conducted | We conducted an online questionnaire survey of college students  by using the Perceived Stress Scale (PSS-10), the Patient Health Questionnaire-9 items  (PHQ-9), and the Self-rating Anxiety Scale (SAS) | The main purpose of this study was to explore the  stress, anxiety, and depression levels of different groups of college students during the  COVID-19 pandemic. | stress, anxiety, and depression levels | • Education departments should attach great importance to the mental health of college students, and it is necessary to provide precise psychological interventions for groups experiencing greater pressure levels and marked anxiety and depression. | 1.The analysis of risk factors is based on only a few simple the survey method used in this study is relatively simple, and the gender differences in depression need further investigation., which may reflect inaccurate mental health outcomes.   1. The psychological state of college students may not only be caused by covid-19, but also because other psychological problems in themselves were aggravated during the epidemic. For example,graduates are more likely to have anxiety than non-graduates |
| 89 | Yi Ding , Xinchen Fu , Rude Liu , Jacqueline Hwang , Wei Hong and Jia Wang | 2021 | China | a total of 492 undergraduate students | Online questionnaire survey, structural equation modeling | The present study aimed to understand the impact of different coping methods endorsed  by Chinese college students during COVID-19 through the examination of the mediating role of  perceived stress. | coping styles; psychological distress;  perceived stress | • Three coping styles were all significantly correlated with psychological distress in Chinese college students during the early stage of the COVID-19 pandemic.  • Adaptive emotion-focused coping was negatively associated with perceived stress and psychological distress.  • Emotion-focused coping was positively associated with perceived stress and distress.  • Individuals who use specific reactive emotion-focused coping strategies more often, such as focusing on  emotions, denial, seeking emotional social support, and disengaging, experience more stress. | 1. the study was based on self-reported questionnaires, which might produce potential biases, although our factor analysis results did not indicate a serious common method bias. 2. we recruited undergraduate students exclusively from two universities in Beijing. It is likely that those who responded to the survey were the individuals who wanted to have a voice and were interested in such a research topic. These college students represented highly educated young people in a metropolitan area in China where the societal and public health resources are relatively abundant. Because of this sampling, the findings of this study might not be generalizable to a population with lower educational attainment and more vulnerable occupational status, or to those in other geographical areas. 3. Third, the survey was conducted fully online. Researchers were unable to reach individuals who might not have had internet access during the early stages of the COVID-19 outbreak; such individuals might have been more vulnerable, and might have perceived higher levels of stress associated with COVID-19. 4. Fourth, the present study only focused on the relations between psychological distress, perceived stress, and three types of coping. There might other factors such as subclinical symptoms and emotional difficulties that might affect students’ self-reporting. Such factors should be further explored in future studies |
| 90 | [YanZhou](https://www.frontiersin.org/people/u/679619), Gunnar Lemmer, Jing Xu and [Winfried Rief](https://www.frontiersin.org/people/u/194894) | 2019 | China and Germany | Data were collected online from   1. groups of Chinese students in China (n = 413) and German students in Germany (n = 416) | Online questionnaire survey | The present study aimed to research on stigma and attitude toward  psychotherapy, and whether these variables are expected to show cross-cultural variations. | stigma, attitudes, psychological help | • Intercultural cooperation should be promoted in order to develop a cross-culturally valid concept of stigma against psychological help that could be used as the basis for intercultural comparison and developing interventions to reduce stigma. | 1. First, the sample was limited to students from China and Germany, so the results cannot be generalized to the general population of these cultures. 2. The compared samples possessed different characteristics (age, gender, and academic degree). Propensity score matching was conducted without including the variable of academic degree, since the category “other” concerning the participant’s academic degrees in the German sample referred to a specific German degree “Staatsexam,” which is equivalent to a bachelor’s degree combined with a master’s degree. Under these circumstances, it is unclear whether measurement invariance occurred due to cultural differences. For future research, it would be better to consider a variable such as “the number of semesters since bachelor” instead of the participants’ “academic degree.” 3. Third, recruitment of a volunteer sample of students may have produced a selection bias, because those who were interested in the topic of seeking psychological help or had experience of psychotherapy may have been more likely to participate. The recruitment of the participants was carried out without a systematic selection process.   4.Therefore, the selfselection of participants, which is a typical disadvantage of online surveys, could be detrimental to the representativeness of the random sample |
| 91 | Liang, Zhengyan;Kang, Derong;Zhang, Minqiang;Xia, Yuanlin;Zeng, Qing | 2021 | China | Chinese postgraduate students from different regions of China | A cross-sectional study through distributing an online  questionnaire;one-way and two-way ANOVA | to understand the mental health status of Chinese postgraduate students during the  COVID-19 pandemic; explore the relationship  between participant characteristics and mental health | anxiety, depression, and social avoidance and distress | • Guiding postgraduate students to correctly understand their mental health status and individual differences in mental tolerance and encouraging postgraduate students to seek help if they experience psychological problems so as to help them adjust their goals and plans according to reality and avoid the development of other problems such as post-traumatic stress disorder;  • establishing an early warning system for the mental health of postgraduate students during the pandemic and improve online and offline psychological counseling service systems;  • considering the characteristics and situation of different postgraduate groups for postgraduate student management to develop targeted mental health education programs and adopting objective measures, so as to improve postgraduate mental health and nurture both physical and mental health to facilitate China’s modernization;  • developing and maintaining conditions to improve the communication between postgraduate students and advisors during the pandemic and create a new postgraduate guidance mode to relieve the psychological problems of postgraduate students | 1. the sampling technique used, which relied on digital infrastructure and voluntary   participation, increases selection bias;  (ii) the study was obtained from one  specific area, did not involve more psychological concepts, such as social distance, loneliness, risk perception, etc   1. this study was a cross-sectional design of   the survey, there was no follow-up period for the participants |
| 92 | Fang-fang Wen , Jian-li Zhu , Han-xue Ye , Lin-yi Li , Zhen Ma , Xiao-xue Wen , Bin Zuo | 2021 | China | undergraduate students  from a university in Hubei province | A cross-sectional study through distributing an online questionnaire | to investigate the parallel mediation role of hope and self efficacy in the relationship between insecurity and stress among university students during the COVID-19  pandemic | insecurity and stress | • Enhancing positive self-beliefs such as hope and self-efficacy helps to buffer the effects of insecurity on stress.  • Physical and psychological exercises that enhance hope can be effective interventions to help university students buffer the impacts of insecurity and alleviate stress during the outbreak.  • Improving positive self-beliefs can help to relieve the pressure of university students during the outbreak.  • University students can improve their self-efficacy by participating in movement based courses including  Pilates and Tai Chi, so as to improve their positive mood and relieve stress. They can also effectively improve their hope levels by setting personal goals and conducting goal-pursuit exercises, which can also contribute to the reduction of insecurity and stress. | 1. the current study mainly focuses on the mediating role of hope and self-efficacy, however based on previous studies, resilience and optimism can also be essential positive psychological variables that help to buffer negative feelings under crisis (Finck et al., 2018; Hou et al., 2017; Poole et al., 2017). Hence, whether these beliefs have impacts on the relationship between perceived insecurity and stress would be worth studying in future research. 2. since this study conducts a cross-sectional survey rather than an experiment, it is hard to come to a cause-and-effect conclusion, which means that the relation between perceived insecurity and stress might be bidirectional. 3. this study examines the mediating model between insecurity and stress among Chinese university students, however the generaliz ability of the findings in other regions remains to be explored. |
| 93 | Xiu-Jiao Lin, Chang-Yuan Zhang, Song Yang, Ming-Lun Hsu, Hui Cheng, Jiang Chen and Hao Yu | 2020 | China | 396 dental undergraduate students at the School of Stomatology, Fujian Medical University, China | A cross-sectional study through distributing an online questionnaire and one-way analysis of variance (ANOVA) | to investigate the amount and sources of stress in dental undergraduate students in Fujian, China, and the factors associated with stress | Stress | • dental schools and educators promote stress coping strategies and modify teaching curricula to reduce students’ stress.  • Stress management efforts such as time management, encouragement from advisors and regular exercise are recommended. | (i) the present findings were based on a single-center, cross sectional study comparing students from different years in the program. It is possible that schools using different methods of teaching may have students with different stress levels (ii)the sample size could limit the power of the regression analyses involving a number of the associated factors. (iii) the current study recruited students from different years of study. Different exams may involve different coping strategies and examination-related self-efficacy. |
